# Aggregation‐Free Organic Dyes Featuring Spiro[dibenzo[3,4:6,7]cyclohepta[1,2‐*b*]quinoxaline‐10,9′‐fluorene] (SDBQX) for Dye‐Sensitized Solar Cells

**DOI:** 10.1002/gch2.201900034

**Published:** 2019-07-03

**Authors:** Jing‐Kun Fang, Mengchen Xu, Xiangyu Hu, Chunxia Wu, Shuang Lu, Hui‐Juan Yu, Xin Bao, Yinglin Wang, Guang Shao, Wei Liu

**Affiliations:** ^1^ Department of Chemistry School of Chemical Engineering Nanjing University of Science and Technology Nanjing 210094 China; ^2^ Center for Advanced Optoelectronic Functional Materials Research and Key Laboratory of UV‐Emitting Materials and Technology of Ministry of Education Northeast Normal University Changchun 130024 China; ^3^ School of Chemistry Sun Yat‐sen University Guangzhou 510275 China; ^4^ Shenzhen Research Institute Sun Yat‐sen University Shenzhen 518057 China; ^5^ Hebei Agricultural University Cangzhou 061100 China; ^6^ Chengde Huakan No.514 Geological Mineral Testing Research Co., Ltd. Chengde 067000 China

**Keywords:** aggregation‐free, chenodeoxycholic acid, dye‐sensitized solar cells, organic dyes

## Abstract

Three novel organic dyes coded as **FHD4‐1**, **FHD4‐2**, and **FHD4‐3** featuring spiro[dibenzo[3,4:6,7]cyclohepta[1,2‐*b*]quinoxaline‐10,9′‐fluorene] (**SDBQX**) moieties are designed to inhibit dye aggregation to improve the performance of dye‐sensitized solar cells (DSSCs). The consistent absorption onsets of **FHD4‐1**, **FHD4‐2**, and **FHD4‐3** in solutions and adsorbed on TiO_2_ films indicate that these dyes are aggregation‐free dyes. Therefore, coadsorption with chenodeoxycholic acid (CDCA) of these three dyes reduces the performance of DSSCs because no inhibition effect for dye aggregation is needed, but, on the contrary, the dye loading amount is reduced after addition of CDCA.

Since it was first reported in 1991, dye‐sensitized solar cells (DSSCs) have attracted wide attention and been well developed.^1^, ^2^, ^3^ Many chemists put their eyes on organic dyes because of their advantages such as low cost, low toxicity, easy structural modification and even efficiency predictable.^4^, ^5^, ^6^, ^7^, ^8^ Many kinds of excellent organic dyes have been developed, such as porphyrin dyes, *N*‐annulated perylene dyes, and many different kinds of rigidified aromatics dyes.^9^, ^10^, ^11^, ^12^ Dyes with D–π–A system is the main type organic dyes, which contains an electron donor (D), an electron acceptor (A), and a π‐conjugated spacer (π) to link the donor and acceptor.^13^, ^14^, ^15^, ^16^ Base on this, dyes with D–A–π–A system were developed by adopting an extra electron deficient spacer to decrease the bandgap and enlarge the absorption range to achieve high power conversion efficiency.^17^, ^18^, ^19^

Dye aggregation is a common phenomenon especially for planar dyes and it is adverse for DSSC performance.^20^ We have reported a series of D–A–π–A organic dyes (**FHD4**, **FHD5**, and **FHD6**) featuring with spiro[dibenzo[3,4:6,7]cyclohepta[1,2‐*b*]quinoxaline‐10,9′‐fluorene] (**SDBQX**) moiety to suppress dye aggregation in DSSCs considering about the fluorenyl moiety is perpendicular to the quinoxaline moiety of **SDBQX** and a 3D structure is realized.^21^ However, dye aggregation was still not be eliminated especially for **FHD5** and **FHD6**. Thus, we designed three organic dyes (**FHD4‐1**, **FHD4‐2**, and **FHD4‐3**) by replacing the electron donor of **FHD4** with electron donor groups adopted long alkyl chains. We expected that thorough suppression of dye aggregation could be observed for these novel organic dyes. The molecular structures of **FHD4‐1**, **FHD4‐2**, and **FHD4‐3** are shown in **Scheme** 1. Their photophysical and electrochemical properties and photovoltaic parameters of DSSCs sensitized by these dyes were investigated systematically.

**Scheme 1:**
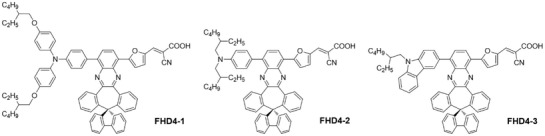
Molecular structures of dyes **FHD4‐1**, **FHD4‐2**, and **FHD4‐3**.

**Scheme** 2 depicts the synthetic routes for **FHD4‐1**, **FHD4‐2**, and **FHD4‐3**. Intermediate **2** could be synthesized by Suzuki cross‐coupling reaction between **1** and (5‐formylfuran‐2‐yl)boronic acid. Then Suzuki cross‐coupling reactions between **2** and (4‐(bis(4‐((2‐ethylhexyl)oxy)phenyl)amino)phenyl)boronic acid, (4‐(bis(2‐ethylhexyl)amino)phenyl)boronic acid, or (9‐(2‐ethylhexyl)‐9*H*‐carbazol‐3‐yl)boronic acid would give the aldehyde **3a**–**3c**, respectively. Finally, cyanoacrylic acid groups were introduced by Knoevenagel reactions between aldehyde **3a**–**3c** and cyanoacetic acid to afford **FHD4‐1**, **FHD4‐2**, and **FHD4‐3**, respectively. **FHD4‐1**, **FHD4‐2**, and **FHD4‐3** were characterized with ^1^H NMR, ^13^C NMR, and HRMS.

**Scheme 2:**
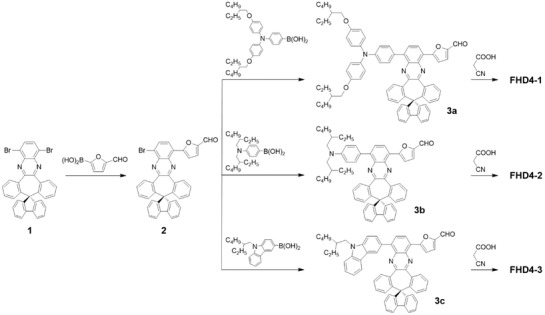
Synthetic routes of **FHD4‐1**, **FHD4‐2**, and **FHD4‐3**.

**Figure** 1 shows the UV–vis absorption spectra of **FHD4‐1**, **FHD4‐2**, and **FHD4‐3** in CH_2_Cl_2_ solutions (2 × 10^−5^ m) and adsorbed on TiO_2_ films. The relevant photophysical data are summarized in **Table** 1. In the solution absorption spectra, all of the three dyes exhibited two prominent peaks at around 410–430 and 490–550 nm. The intense absorption bands in short wavelength region could be assigned to the π–π* transition.^21^ The absorption peaks in long wavelength region corresponding to the intramolecular charge transfer (ICT) from the electron donor to the electron acceptor were observed at 531, 545, and 490 nm for **FHD4‐1**, **FHD4‐2**, and **FHD4‐3**, respectively.^21^ Broader spectral coverages were observed for **FHD4‐1** and **FHD4‐2** compared with **FHD4‐3**. Similar molar extinction coefficients (ε) were observed for these three dyes, the high ε values guaranteed their good light harvesting capabilities.^22^ Based on the molecular exciton theory, dye aggregation would lead to a shift of absorption spectrum.^20^ As for **FHD4**, an obvious bathochromic shift was observed for absorption spectrum on TiO_2_ film compared with in solution, which suggested that the existence of *J*‐aggregation for **FHD4** molecules on the surface of TiO_2_.^21^ The absorption onsets of **FHD4‐1**, **FHD4‐2**, and **FHD4‐3** in solutions and adsorbed on TiO_2_ films had good consistency and no apparent shifts were observed. It demonstrates that dye aggregation could be suppressed efficiently by adopting donors with long alkyl chains, and hence **FHD4‐1**, **FHD4‐2**, and **FHD4‐3** are confirmed to be aggregation free dyes and their well performance in DSSC could be expected due to aggregation is a key adverse factor for DSSC performance.

**Figure 1:**
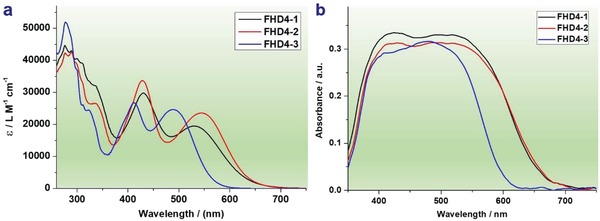
UV–vis absorption spectra of **FHD4‐1**, **FHD4‐2**, and **FHD4‐3** a) in solutions and b) adsorbed on TiO_2_ films.

**Table 1:** Photophysical and electrochemical data of **FHD4‐1**, **FHD4‐2**, and **FHD4‐3**

Dye	λ_max_ [nm]^a)^	ε [10^4^ m ^−1^ cm^−1^]^a)^	*E*_ox_ [V]^b)^	*E*_0‐0_ [V]^c)^	*E*_red_ [V]^d)^	Bandgap [eV]^e)^
**FHD4‐1**	531 429	2.02 3.03	0.86	1.76	−0.90	2.14
**FHD4‐2**	545 429	2.36 3.35	0.97	1.78	−0.81	2.42
**FHD4‐3**	490 411	2.46 2.64	1.37	2.04	−0.67	2.63

^a)^ In CH_2_Cl_2_ solution

^b)^ First oxidation potentials (*E*_ox_) (vs NHE) were calibrated with ferrocene (0.63 V vs NHE)

^c)^ E_0‐0_ transition energy was calculated from optical absorption onset

^d)^ *E*_red_ = *E*_ox_ – *E*_0–0_

^e)^ DFT/B3LYP calculated values.

The redox potentials of **FHD4‐1**, **FHD4‐2**, and **FHD4‐3** were determined by cyclic voltammetry to evaluate the feasibilities of the electron injection and dye regeneration. The cyclic voltammograms are shown in **Figure** 2a and the corresponding data are summarized in Table 1. The first oxidation potentials (*E*_ox_) of dyes **FHD4‐1** (0.86 V), **FHD4‐2** (0.97 V), and **FHD4‐3** (1.37 V) were sufficiently more positive than the I_3_^−^/I^−^ redox potential (0.4 V vs normal hydrogen electrode, NHE), so efficient dye regeneration of the oxidized dyes by the I_3_^−^/I^−^ electrolyte could be expected.^23^ The energy band diagrams of the dyes were showed in Figure 2b. The optic bandgap energies (*E*_0–0_) of **FHD4‐1** (1.76 V) and **FHD4‐2** (1.78 V) were much lower than that of **FHD4‐3** (2.04 V), resulting in broader adsorption spectra for **FHD4** and **FHD5**, which is beneficial for their light harvesting capabilities. *E*_red_ can be calculated from *E*_ox_ – *E*_0‐0_. The *E*_red_ of dyes **FHD4‐1** (−0.90 V), **FHD4‐2** (−0.81 V), and **FHD4‐3** (−0.67 V) were negative than the conduction band (CB) edge of TiO_2_ (−0.5 V vs NHE). The potential difference between these dyes and the CB edge of TiO_2_ are getting smaller in the sequence of **FHD4‐1**, **FHD4‐2**, and **FHD4‐3**. The latter one might not enough to guarantee the thermodynamic feasibility of charge injections from excited dye molecules to the CB of TiO_2_, which might restrict the photovoltaic performances for DSSCs.^24^

**Figure 2:**
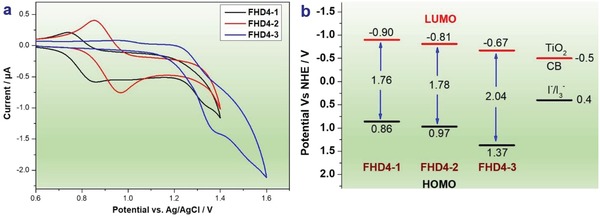
a) CV plots and b) energy band diagram of **FHD4‐1**, **FHD4‐2**, and **FHD4‐3**.

The optimized geometrical structures and electron distributions of **FHD4‐1**, **FHD4‐2**, and **FHD4‐3** were simulated by density functional theory (DFT) calculations with the B3LYP exchange correlation functional under the 6‐31G (d,p) basis set implemented in the Gaussian 09 program. The simulated electron distributions in highest occupied molecular orbital (HOMO) and lowest unoccupied molecular orbital (LUMO) levels of the dyes are shown in **Figure** 3, while the isodensity surface values were fixed at 0.02. As shown in their molecular orbital profiles, the electrons mainly distributed over the triphenylamine moiety with a little contribution over the quinoxaline core for the HOMO of **FHD4‐1**, and distributed over the whole molecules for the HOMOs of **FHD4‐2** and **FHD4‐3**. For the LUMOs, the electrons located on the quinoxaline core and electron acceptors (furanylacrylic acid moiety) for all of these three dyes. The bandgaps between HOMOs and LUMOs according to DFT calculations (2.14, 2.42, and 2.63 V for **FHD4‐1, FHD4‐2**, and **FHD4‐3**, respectively) exhibited consistent trend with the *E*_0–0_ values obtained from onsets of the absorption spectra. **Figure** 4 shows the calculated dihedral angles between quinoxaline of **SDBQX** moieties and the aromatic rings connected with quinoxalines in the optimized structures of **FHD4‐1**, **FHD4‐2**, and **FHD4‐3**. It is clear that the dihedral angles between quinoxaline moieties and electron donating groups are very similar (39.6°, 38.5°, and 41.2° for **FHD4‐1, FHD4‐2**, and **FHD4‐3**, respectively), and the dihedral angles between quinoxaline moieties and furan rings are almost same (6.3°, 6.4°, and 6.8° for **FHD4‐1, FHD4‐2**, and **FHD4‐3**, respectively). Based on the dihedral angle data, quinoxaline moieties showed an approximate coplanar geometry with the furan rings for **FHD4‐1**, **FHD4‐2**, and **FHD4‐3**. The big dihedral angles between quinoxaline moieties and electron donating groups is beneficial to ICT, and hence enlarging the absorption spectra range, which could enhance the light harvesting capabilities of these dyes.^25^, ^26^

**Figure 3:**
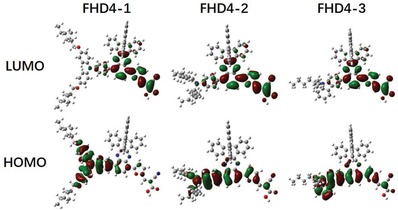
HOMO and LUMO electron distributions of **FHD4‐1**, **FHD4‐2**, and **FHD4‐3**.

**Figure 4:**
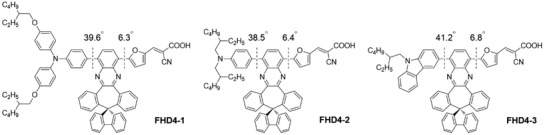
Calculated dihedral angles in optimized structures of **FHD4‐1**, **FHD4‐2**, and **FHD4‐3**.

The photovoltaic performances of the DSSCs based on **FHD4‐1**, **FHD4‐2**, and **FHD4‐3** with or without chenodeoxycholic acid (CDCA) were evaluated under illumination simulated AM 1.5G irradiation (100 mW cm^−2^). **Figure** 5 shows the photocurrent density–voltage (*J*–*V*) curves and the incident photon‐to‐current conversion efficiencies (IPCE) characteristic plots. The detailed corresponding photoelectrode chemical data of short‐circuit current density (*J*_sc_), open‐circuit voltage (*V*_oc_), fill factor (FF), and power conversion efficiency (η) are listed in **Table** 2. The DSSCs based on **FHD4‐1**, **FHD4‐2**, and **FHD4‐3** exhibited high short‐circuit current density (*J*_sc_ = 10.44, 12.94, and 11.00 mA cm^−2^, respectively) as well as high open‐circuit photovoltage (*V*_oc_ = 0.72, 0.68, and 0.71 V, respectively), and hence good power conversion efficiency (η = 4.54%, 5.16%, and 4.87%, respectively) was observed for them. The high *J*_sc_ value of DSSCs based on **FHD4‐2** guaranteed its highest power conversion efficiency among these three dyes even if a little lower *V*_oc_ was observed for it. The IPCE plots were measured to further study the *J*_sc_ of the DSSCs based on **FHD4‐1**, **FHD4‐2**, and **FHD4‐3**. As shown in Figure 5b, the devices based on these three dyes exhibited broad spectral responses, which were well consistent with the absorption spectra on TiO_2_ films (Figure 1b). Low response intensity for **FHD4‐1** and narrow response range for **FHD4‐3** resulting in there lower *J*_sc_ values compared with **FHD4‐2**.

**Figure 5:**
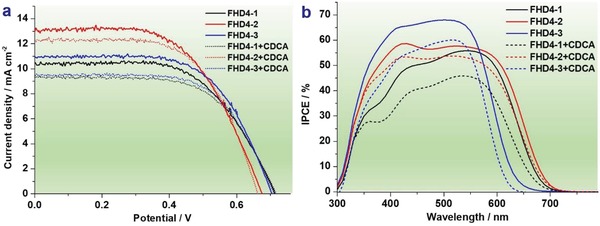
a) *J*–*V* curves and b) IPCE plots of DSSCs based on **FHD4‐1**, **FHD4‐2**, and **FHD4‐3**.

**Table 2:** Photovoltaic performance parameters of DSSCs based on **FHD4‐1**, **FHD4‐2**, and **FHD4‐3**

Dye	CDCA [×10^−3^ m]	*J*_sc_ [mA cm^−2^]	*V*_oc_ [V]	FF [%]	η [%]	Γ [M cm^−2^]
**FHD4‐1**	0	10.44 ± 0.45	0.72 ± 0.01	60.86 ± 1.51	4.54 ± 0.06	1.41 × 10^−7^
	3	9.36 ± 0.47	0.72 ± 0.01	61.97 ± 0.77	4.17 ± 0.22	1.13 × 10^−7^
**FHD4‐2**	0	12.94 ± 0.42	0.68 ± 0.01	59.06 ± 1.42	5.16 ± 0.03	1.31 × 10^−7^
	3	12.36 ± 0.36	0.67 ± 0.02	61.79 ± 1.21	5.08 ± 0.15	1.08 × 10^−7^
**FHD4‐3**	0	11.00 ± 0.25	0.71 ± 0.02	62.67 ± 0.86	4.87 ± 0.02	1.79 × 10^−7^
	3	9.54 ± 0.24	0.71 ± 0.01	63.93 ± 0.87	4.34 ± 0.07	1.32 × 10^−7^

Typically, the addition of CDCA would improve the power conversion efficiency because it could obstruct the dye aggregation on TiO_2_ film.^27^ Similar effects were observed for **FHD4**, **FHD5**, and **FHD6** in our previous work.^21^ However, after coadsorption with CDCA, lower efficiencies were observed for analogues **FHD4‐1**, **FHD4‐2**, and **FHD4‐3** (4.17%, 5.08%, and 4.34%, respectively) featuring with long alkyl chains mainly due to the reduction of *J*_sc_ values. As we discussed above, **FHD4‐1**, **FHD4‐2**, and **FHD4‐3** are aggregation free dyes which means no auxiliary additive such as CDCA is necessary to prevent the dye aggregation. Moreover, the addition of CDCA could reduce the chance of dye adsorption,^28^ so lower *J*_sc_ values could be expected which is well coincident with our study. It could also explain the lower response intensity of IPCE for the DSSCs coadsorbed with CDCA. To examine this guess, dye loading amount (*Γ*) on TiO_2_ of **FHD4‐1**, **FHD4‐2**, and **FHD4‐3** with or without CDCA were measured. As shown in Table 2, the *Γ* values of **FHD4‐1**, **FHD4‐2**, and **FHD4‐3** decreased from 1.41 × 10^−7^, 1.31 × 10^−7^, 1.79 × 10^−7^ m cm^−2^ (no CDCA) to 1.13 × 10^−7^, 1.08 × 10^−7^, 1.32 × 10^−7^ m cm^−2^ (with 3 × 10^−3^ m CDCA), respectively. It is clear that dye loading amount reduced after coadsorption with CDCA for all of these three dyes as we expected above.

In summary, three novel organic dyes featuring with **SDBQX** moieties (**FHD4‐1**, **FHD4‐2**, and **FHD4‐3**) were designed and synthesized. These dyes were characterized with ^1^H NMR, ^13^C NMR, and HRMS. The absorption onsets of **FHD4‐1**, **FHD4‐2**, and **FHD4‐3** in solutions and adsorbed on TiO_2_ films had good consistency and no apparent shifts were observed. It demonstrates that **FHD4‐1**, **FHD4‐2**, and **FHD4‐3** are aggregation free dyes. Good power conversion efficiency was achieved for **FHD4‐1**, **FHD4‐2**, and **FHD4‐3** (4.54%, 5.16%, and 4.87%, respectively). Coadsorption with CDCA reduced the efficiency to 4.17%, 5.08%, and 4.34%, respectively. It was attributed to the reduce of dye loading amount and hence the *J*_sc_ value after addition of CDCA. It was proved that no auxiliary additive such as CDCA is necessary for aggregation free dyes **FHD4‐1**, **FHD4‐2**, and **FHD4‐3** to prevent dye aggregation.

## Conflict of Interest

The authors declare no conflict of interest.

## Supporting information

Supplementary

## References

[1] B. O'Regan , M. Grätzel , Nature 1991, 353, 737.

[2] F. Bella , C. Gerbaldi , C. Barolo , M. Gratzel , Chem. Soc. Rev. 2015, 44, 3431. 25864577 10.1039/c4cs00456f

[3] A. Blaszczyk , Dyes Pigm. 2018, 149, 707.

[4] V. Venkatraman , R. Raju , S. P. Oikonomopoulos , B. K. Alsberg , J. Cheminf. 2018, 10, 18. 10.1186/s13321-018-0272-0 PMC5882482 29616364

[5] S. Chaurasia , J. T. Lin , Chem. Rec. 2016, 16, 1311. 27114164 10.1002/tcr.201500288

[6] X. Ying , Y. Liu , L. Ling , M. Xu , B. Bao , X. Bao , A. Pang , G. Shao , Y. Zhang , J. K. Fang , Sol. RRL 2019, 3, 1900066.

[7] Z. S. Huang , H. Meier , D. R. Cao , J. Mater. Chem. C 2016, 4, 3662.

[8] H. Meier , Z. S. Huang , D. R. Cao , J. Mater. Chem. C 2017, 5, 9828.

[9] M. Urbani , M. Gratzel , M. K. Nazeeruddin , T. Torres , Chem. Rev. 2014, 114, 12330. 25495339 10.1021/cr5001964

[10] Z. Y. Yao , M. Zhang , H. Wu , L. Yang , R. Z. Li , P. Wang , J. Am. Chem. Soc. 2015, 137, 3799. 25742441 10.1021/jacs.5b01537

[11] J. Liu , Y. Ren , M. Zhang , X. Dong , Sol. RRL 2018, 2, 1800119.

[12] S. Chaurasia , C. J. Liang , Y. S. Yen , J. T. Lin , J. Mater. Chem. C. 2015, 3, 9765.

[13] N. Balis , A. A. Zaky , D. Perganti , A. Kaltzoglou , L. Sygellou , F. Katsaros , T. Stergiopoulos , A. G. Kontos , P. Falaras , ACS Appl. Energy Mater. 2018, 1, 6161.

[14] J. K. Fang , T. X. Sun , Y. Tian , Y. J. Zhang , C. F. Jin , Z. M. Xu , Y. Fang , X. Y. Hu , H. B. Wang , Mater. Chem. Phys. 2017, 195, 1.

[15] H. B. Wang , B. Z. Bao , X. Y. Hu , J. K. Fang , Electrochim. Acta 2017, 250, 278.

[16] A. Mahmood , Sol. Energy. 2016, 123, 127.

[17] Y. Z. Wu , W. H. Zhu , S. M. Zakeeruddin , M. Gratzel , ACS Appl. Mater. Interfaces 2015, 7, 9307. 25899976 10.1021/acsami.5b02475

[18] H. B. Zhu , W. Q. Li , Y. Z. Wu , B. Liu , S. Q. Zhu , X. Li , H. Agren , W. H. Zhu , ACS Sustainable Chem. Eng. 2014, 2, 1026.

[19] S. R. Li , C. P. Lee , C. W. Liao , W. L. Su , C. T. Li , K. C. Ho , S. S. Sun , Tetrahedron 2014, 70, 6276.

[20] L. Zhang , J. M. Cole , J. Mater. Chem. A 2017, 5, 19541.

[21] M. Xu , X. Hu , Y. Zhang , X. Bao , A. Pang , J. K. Fang , ACS Appl. Energy Mater. 2018, 1, 2200.

[22] J. K. Fang , X. Yu , X. Yang , W. F. Li , D. L. An , Chin. J. Org. Chem. 2012, 32, 1261.

[23] T. Hua , Z. S. Huang , K. Cai , L. Y. Wang , H. Tang , H. Meier , D. R. Cao , Electrochim. Acta 2019, 302, 225.

[24] A. Karuppasamy , K. Stalindurai , J. D. Peng , K. C. Ho , C. Ramalingan , Electrochim. Acta 2018, 268, 347.

[25] J. K. Fang , D. L. An , K. Wakamatsu , T. Ishikawa , T. Iwanaga , S. Toyota , S. Akita , D. Matsuo , A. Orita , J. Otera , Tetrahedron 2010, 66, 5479.

[26] J. K. Fang , D. L. An , K. Wakamatsu , T. Ishikawa , T. Iwanaga , S. Toyota , D. Matsuo , A. Orita , J. Otera , Tetrahedron Lett. 2010, 51, 917.

[27] B. S. Arslan , E. Guzel , T. Kaya , V. Durmaz , M. Keskin , D. Avci , M. Nebioglu , I. Sisman , Dyes Pigm. 2019, 164, 188.

[28] J. K. Fang , X. Y. Hu , M. C. Xu , H. B. Wang , Y. J. Zhang , C. F. Jin , X. Zhong , Electrochim. Acta 2017, 253, 572.

