# Supplementary material for: Aggregation‐Free Organic Dyes Featuring Spiro[dibenzo[3,4:6,7]cyclohepta[1,2‐b]quinoxaline‐10,9′‐fluorene] (SDBQX) for Dye‐Sensitized Solar Cells
Source: Glob Chall. 2019 Jul 3;3(10):1900034. doi: 10.1002/gch2.201900034 (PMC6777215; doi:10.1002/gch2.201900034)
Supplement: Supplementary file 1 — Supplementary [file GCH2-3-1900034-s001.pdf]

Copyright WILEY-VCH Verlag GmbH & Co. KGaA, 69469 Weinheim, Germany, 2019.

Global Challenges
Open Access

Supporting Information

for *Global Challenges,* DOI: 10.1002/gch2.201900034

Aggregation-Free Organic Dyes Featuring Spiro[dibenzo[3,4:6,7]cyclohepta[1,2-*b*]quinoxaline-10,9'-fluorene] (SDBQX) for Dye-Sensitized Solar Cells

Jing-Kun Fang, Mengchen Xu, Xiangyu Hu, Chunxia Wu, Shuang Lu, Hui-Juan Yu, Xin Bao, Yinglin Wang,* Guang Shao,* and Wei Liu*

Copyright WILEY-VCH Verlag GmbH & Co. KGaA, 69469 Weinheim, Germany, 2019.

Supporting Information

Aggregation Free Organic Dyes Featuring with Spiro[dibenzo[3,4:6,7]cyclohepta[1,2-*b*]quinoxaline-10,9'-fluorene] (SDBQX) for Dye-Sensitized Solar Cells

Jing-Kun Fang, Mengchen Xu, Xiangyu Hu, Chunxia Wu, Shuang Lu, Hui-Juan Yu, Xin Bao, Yinglin Wang,* Guang Shao,* and Wei Liu*

Contents

1. Materials and Instruments

2. Experimental Protocols and Compound Data

3. NMR Spectra

4. Mass Spectra

5. Fabrication of DSSCs

6. Supporting References

1. Materials and Instruments

All reagents obtained from commercial sources were used as received, unless otherwise noted. All organic solvents were purified according to the standard methods. Column chromatography were carried out on chromatography silica gel (300 ~ 400 mesh).

Melting points were measured on an X-4A apparatus. ^{1}H NMR and ^{13}C NMR spectra were recorded at room temperature on Bruker AVANCE III 500 instruments and calibrated with tetramethylsilane (TMS) as an internal reference. HRMS spectra were recorded on an ESI-Q-TOF mass spectrometer (Bruker Daltonics, ESI-Q-TOFmaXis 4G). UV-Vis absorption spectra were recorded by Analytik Jena SPECORD 50 PLUS (in solution) and Hitachi UH 4150 UV-visible-NIR spectrophotometer (on TiO_2 film). Cyclic voltammetry measurements of dyes were carried out with CHI660E electrochemical workstation in tetrahydrofuran (THF) (1.0×10^{-3} M) containing 0.1 M Bu_4NPF_6 as the supporting electrolyte, and a three-electrode system (glassy carbon as the working electrode, platinum wire as the counter electrode, and Ag/AgCl as reference electrode). Ferrocene was used as the external standard. The incident photon-to-current efficiencies (IPCE) were measured with mono-chromatic incident light under 100 mW cm^{-2} with bias light in DC mode (PECCELL PEC-S20, using a 1KW Xe Arc lamp). The current density-voltage (*J*-*V*) characteristics of the DSSCs were measured by recording *J*-*V* curves using a Keithley 2600 source meter using simulated 1.5 AM sunlight with an output power of 100 mW cm^{-2} with a solar light simulator (PECCELL PEC-L15, using a 1KW Xe Arc lamp) without a mask. Electrochemical impedance spectroscopy (EIS) were measured on Zahner PP211 electrochemical workstation under dark station under a forward bias of -0.70 V with a frequency range of 0.1 Hz ~ 1 MHz.

2. Experimental Protocols and Compound Data

All reactions were carried out under an atmosphere of nitrogen unless otherwise noted and monitored by thin layer chromatography (TLC). 1,4-dibromospiro[dibenzo[3,4:6,7]cyclohepta[1,2-*b*]quinoxaline-10,9'-fluorene] (**1**) was synthesized according to the literature.[S1]

5-(1-bromospiro[dibenzo[3,4:6,7]cyclohepta[1,2-*b*]quinoxaline-10,9'-fluoren]-4-yl)furan-2-carbaldehyde (2)

To a flask were added **1** (903 mg, 1.50 mmol), (5-formylfuran-2-yl)boronic acid (140 mg, 1.00 mmol), $Pd(PPh_3)_4$ (69 mg, 0.06 mmol), Na_2CO_3 (318 mg, 3.00 mmol), THF (71 mL) and H_2O (18 mL), and the mixture was heated to reflux overnight. Then the reaction mixture was poured into aqueous NH_4Cl and extracted with CH_2Cl_2. The organic layer was washed with saturated brine and dried over Na_2SO_4. After filtration, solvents were removed by rotary evaporation. The crude product was subjected to column chromatography (SiO_2; eluent, PE/CH_2Cl_2, 2:1) to give **2** as yellow powder (120 mg, 20% yield). M.p: 170-173℃. ^{1}H NMR (500 MHz, $CDCl_3$) δ (ppm): 9.74 (s, 1H), 8.63 (d, J = 7.5 Hz, 1H), 8.46 (d, J = 8.0 Hz, 1H), 8.26 (d, J = 8.0 Hz, 2H), 7.97 (d, J = 4.0 Hz, 1H), 7.76 (d, J = 7.5 Hz, 2H), 7.51-7.47 (m, 2H), 7.38 (d, J = 3.5 Hz, 1H), 7.34 (t, J = 7.5 Hz, 2H), 7.27-7.21 (m, 4H), 7.06 (t, J = 8.0 Hz, 2H), 6.76 (br, 2H). ^{13}C NMR (126 MHz, $CDCl_3$) δ (ppm): 177.37, 155.17, 153.54, 151.50, 149.31, 146.07, 145.71, 140.04, 139.40, 138.70, 137.52, 137.03, 134.04, 133.42, 130.14, 129.90, 128.65, 128.55, 128.05, 127.95, 127.88, 127.54, 127.19, 126.80, 126.09, 123.89, 120.49, 116.09, 66.49.

General procedures for the preparation of intermediates 3a-3c.

To a flask were added **2** (130 mg, 0.21 mmol), the corresponding arylboronic acid (0.50 mmol), $Pd(PPh_3)_4$ (18 mg, 0.016 mmol), Na_2CO_3 (67 mg, 0.63 mmol), DMF (7 mL) and H_2O (2 mL), and the mixture was heated to reflux overnight. Then the reaction mixture was poured into aqueous NH_4Cl and extracted with CH_2Cl_2. The organic layer was washed with saturated

brine and dried over Na_2SO_4. After filtration, solvents were removed by rotary evaporation. The crude product was subjected to column chromatography (SiO_2; eluent, PE/CH_2Cl_2, 1:1) to give **3a-3c**, respectively.

5-(1-(4-(bis(4-((2-ethylhexyl)oxy)phenyl)amino)phenyl)spiro[dibenzo[3,4:6,7]cyclohepta[1,2-*b*]quinoxaline-10,9'-fluoren]-4-yl)furan-2-carbaldehyde (3a).

Red powder, 94% yield. M.p: 98-100℃. ^1H NMR (500 MHz, $CDCl_3$) δ (ppm): 9.73 (s, 1H), 8.65 (d, *J* = 8.0 Hz, 1H), 8.43 (d, *J* = 7.5 Hz, 1H), 8.29 (d, *J* = 7.5 Hz, 1H), 8.00 (d, *J* = 7.5 Hz, 1H), 7.97 (m, 1H), 7.75 (t, *J* = 8.0 Hz, 4H), 7.49 (t, *J* = 7.5 Hz, 1H), 7.39-7.36 (m, 2H), 7.32 (t, *J* = 7.5 Hz, 2H), 7.24-7.16 (m, 4H), 7.13 (d, *J* = 7.0 Hz, 4H), 7.06-7.05 (m, 4H), 6.85 (d, *J* = 7.0 Hz, 4H), 6.77 (br, 2H), 3.82 (d, *J* = 5.5 Hz, 4H), 1.74-1.69 (m, 2H), 1.52-1.22 (m, 16H), 0.95-0.90 (m, 12H). ^{13}C NMR (126 MHz, $CDCl_3$) δ (ppm): 177.27, 156.37, 155.99, 152.23, 151.81, 151.26, 149.48, 148.93, 145.77, 145.56, 141.72, 140.31, 140.06, 139.49, 138.74, 138.21, 137.82, 133.92, 133.44, 131.60, 129.49, 129.33, 129.03, 128.51, 128.45, 128.19, 127.96, 127.80, 127.15, 127.02, 125.11, 124.34, 120.40, 119.06, 115.52, 115.30, 70.73, 66.53, 39.45, 30.53, 29.09, 23.87, 23.03, 14.07, 11.12.

5-(1-(4-(bis(2-ethylhexyl)amino)phenyl)spiro[dibenzo[3,4:6,7]cyclohepta[1,2-*b*]quinoxaline-10,9'-fluoren]-4-yl)furan-2-carbaldehyde (3b).

Red powder, 80% yield. M.p: 69-72℃. ^1H NMR (500 MHz, $CDCl_3$) δ (ppm): 9.72 (s, 1H), 8.65 (d, *J* = 8.0 Hz, 1H), 8.51 (d, *J* = 7.5 Hz, 1H), 8.31 (d, *J* = 7.5 Hz, 1H), 8.02 (d, *J* = 8.0 Hz, 1H), 7.97 (d, *J* = 3.4 Hz, 1H), 7.87 (d, *J* = 9.0 Hz, 2H), 7.75 (d, *J* = 7.5 Hz, 2H), 7.49 (dt, *J* = 2.0, 7.5 Hz, 1H), 7.39 (d, *J* = 3.5 Hz, 1H), 7.37-7.32 (m, 3H), 7.24-7.14 (m, 4H), 7.06-7.05 (m, 2H), 6.80 (d, *J* = 8.5 Hz, 4H), 3.37-3.23 (m, 4H), 1.89 (m, 2H), 1.43 -1.30 (m, 16H), 0.93-0.88 (m, 12H). ^{13}C NMR (126 MHz, $CDCl_3$) δ (ppm): 177.16, 156.64, 152.00, 151.50, 151.13, 149.51, 148.29, 145.71, 145.45, 142.12, 140.04, 139.50, 138.89, 138.29, 137.98, 133.91, 133.57, 131.94, 129.38, 128.68, 128.46, 128.40, 127.93, 127.76, 127.65, 127.16,

124.24, 124.06, 120.37, 115.20, 112.04, 66.54, 56.28, 36.83, 30.64, 28.70, 23.87, 23.20, 14.09, 10.68.

5-(1-(9-(2-ethylhexyl)-9H-carbazol-3-yl)spiro[dibenzo[3,4:6,7]cyclohepta[1,2-*b*]quinoxaline-10,9'-fluoren]-4-yl)furan-2-carbaldehyde (3c).

Orange red powder, 95% yield. M.p: 155-157℃. ^{1}H NMR (500 MHz, $CDCl_3$) δ (ppm): 9.76 (s, 1H), 8.80 (s, 1H), 8.74 (d, J = 8.0 Hz, 1H), 8.56 (d, J = 8.0 Hz, 1H), 8.33 (d, J = 8.0 Hz, 1H), 8.19 (d, J = 7.5 Hz, 2H), 8.04-8.02 (m, 2H), 7.74 (d, J = 7.5 Hz, 2H), 7.54-7.41 (m, 5H), 7.33 (t, J = 7.5 Hz, 2H), 7.30-7.21 (m, 4H), 7.17 (d, J = 7.5 Hz, 1H), 7.05-7.12 (m, 3H), 6.81 (br, 2H), 4.27-4.18 (m, 2H), 2.15-2.10 (m, 1H), 1.43-1.27 (m, 8H), 0.94 (t, J = 7.5 Hz, 3H), 0.88 (t, J = 7.5 Hz, 3H). ^{13}C NMR (126 MHz, $CDCl_3$) δ (ppm): 177.29, 156.37, 152.19, 151.93, 151.30, 149.53, 145.79, 145.51, 142.54, 141.37, 140.91, 140.05, 139.65, 138.79, 138.22, 137.83, 134.00, 133.41, 130.03, 129.50, 128.87, 128.52, 128.45, 128.19, 127.95, 127.81, 127.17, 125.68, 125.22, 124.30, 123.22, 122.82, 120.39, 120.19, 119.06, 115.58, 109.16, 108.60, 66.56, 47.53, 39.43, 31.00, 28.82, 24.34, 23.04, 14.02, 10.86.

General procedures for the preparation of dyes FHD4-1, FHD4-2 and FHD4-3.

To a flask were added **3a-3c** (0.15 mmol), 2-cyanoacetic acid (26 mg, 0.30 mmol), ammonium acetate (5 mg, 0.06 mmol), acetic acid (3 mL) and toluene (9 mL), and the mixture was heated to reflux for 4 h. Then the reaction mixture was poured into water and extracted with CH_2Cl_2. The organic layer was washed with saturated brine and dried over Na_2SO_4. After filtration, solvents were removed by rotary evaporation. The crude product was subjected to column chromatography (SiO_2; eluent, CH_2Cl_2/MeOH, 30:1 to 10:1) to give **FHD4-1**, **FHD4-2** and **FHD4-3**, respectively.

3-(5-(1-(4-(bis(4-((2-ethylhexyl)oxy)phenyl)amino)phenyl)spiro[dibenzo[3,4:6,7]cyclohepta[1,2-*b*]quinoxaline-10,9'-fluoren]-4-yl)furan-2-yl)-2-cyanoacrylic acid (FHD4-1).

Red powder, 96% yield. M.p: 260-263℃. ^{1}H NMR (500 MHz, DMSO-d_6) δ (ppm): 8.54 (d, J = 8.0 Hz, 1H), 8.29 (d, J = 7.5 Hz, 1H), 8.23 (d, J = 7.5 Hz, 1H), 8.04 (d, J = 8.0 Hz, 1H), 7.94 (d, J = 7.5 Hz, 2H), 7.87 (s, 1H), 7.85 (d, J = 4.0 Hz, 1H), 7.72 (d, J = 8.5 Hz, 2H), 7.56 (t, J = 7.5 Hz, 1H), 7.37-7.33 (m, 4H), 7.30 (d, J = 3.0 Hz, 1H), 7.25 (t, J = 8.0 Hz, 1H), 7.12 (d, J = 8.0 Hz, 1H), 7.08 (d, J = 8.0 Hz, 1H), 7.05 (d, J = 8.5 Hz, 4H), 6.91 (d, J = 8.5 Hz, 4H), 6.84 (d, J = 8.5 Hz, 2H), 6.65 (br, 4H), 3.82 (d, J = 5.5 Hz, 4H), 1.69-1.62 (m, 2H), 1.48-1.32 (m, 8H), 1.29-1.26 (m, 8H), 0.90-0.85 (m, 12H). ^{13}C NMR (126 MHz, DMSO-d_6) δ (ppm): 153.12, 151.34, 151.26, 148.65, 148.52, 145.09, 145.01, 140.85, 140.52, 140.25, 139.56, 138.96, 137.79, 137.49, 137.28, 133.77, 133.38, 130.43, 129.84, 129.65, 128.70, 128.41, 128.24, 127.85, 127.66, 127.54, 127.48, 127.31, 125.84, 125.10, 123.08, 122.40, 122.01, 121.04, 120.08, 119.06, 115.88, 109.60, 108.84, 65.93, 46.67, 38.76, 30.20, 28.09, 23.63, 22.53, 13.84, 10.63. HRMS (ESI/Q-TOF, m/z): Calcd for $[M]^+$ $C_{75}H_{68}N_4O_5^+$: 1104.5184, found: 1104.5185.

3-(5-(1-(4-(bis(2-ethylhexyl)amino)phenyl)spiro[dibenzo[3,4:6,7]cyclohepta[1,2-*b*]quinoxaline-10,9'-fluoren]-4-yl)furan-2-yl)-2-cyanoacrylic acid (FHD4-2).

Dark red powder, 94% yield. M.p: 210-213℃. ^{1}H NMR (500 MHz, DMSO-d_6) δ (ppm): 8.57 (d, J = 8.0 Hz, 1H), 8.36 (d, J = 7.5 Hz, 1H), 8.30 (d, J = 7.5 Hz, 1H), 8.05 (d, J = 8.0 Hz, 1H), 8.02 (s, 1H), 7.96-7.91 (m, 3H), 7.78 (d, J = 8.0 Hz, 2H), 7.60 (t, J = 7.5 Hz, 1H), 7.48 (s, 1H), 7.37-7.34 (m, 4H), 7.27 (t, J = 7.5 Hz, 1H), 7.13 (d, J = 8.0 Hz, 2H), 7.11 (d, J = 8.0 Hz, 2H), 6.78 (d, J = 8.0 Hz, 2H), 6.64 (br, 2H), 3.27 (m, 4H), 1.77 (m, 2H), 1.25 (m, 16H), 0.84 (m, 12H). ^{13}C NMR (126 MHz, $CDCl_3$) δ (ppm): 157.30, 151.62, 151.07, 149.53, 148.14, 147.32, 145.30, 141.50, 139.95, 139.13, 138.68, 138.10, 138.02, 133.92, 133.60, 131.95, 129.20, 129.11, 129.00, 128.16, 127.79, 127.38, 127.07, 124.19, 123.86, 120.20, 116.79, 111.94, 66.43, 56.07, 36.75, 30.53, 28.59, 23.73, 23.19, 14.09, 10.59. HRMS (ESI/Q-TOF, m/z): Calcd for $[M]^+$ $C_{63}H_{60}N_4O_3^+$: 920.4660, found: 920.4661.

2-Cyano-3-(5-(1-(9-(2-ethylhexyl)-9H-carbazol-3-yl)spiro[dibenzo[3,4:6,7]cyclohepta[1,2-*b*]quinoxaline-10,9'-fluoren]-4-yl)furan-2-yl)acrylic acid (FHD4-3).

Orange powder, 96% yield. M.p: >300℃. ^{1}H NMR (500 MHz, DMSO-d_6) δ (ppm): 8.85 (s, 1H), 8.67 (d, *J* = 8.0 Hz, 1H), 8.44 (d, *J* = 7.5 Hz, 1H), 8.33 (d, *J* = 7.5 Hz, 1H), 8.27 (d, *J* = 7.5 Hz, 1H), 8.20 (d, *J* = 7.5 Hz, 1H), 8.00 (d, *J* = 8.5 Hz, 1H), 7.95-7.93 (m, 4H), 7.65 (d, *J* = 8.5 Hz, 1H), 7.61-7.57 (m, 2H), 7.48 (t, *J* = 8.0 Hz, 1H), 7.41-7.34 (m, 4H), 7.29-7.19 (m, 4H), 7.13 (d, *J* = 8.0 Hz, 1H), 7.08 (br, 1H), 7.06 (d, *J* = 8.0 Hz, 1H), 6.63 (br, 2H), 4.27 (d, *J* = 6.5 Hz, 2H), 2.03-1.99 (m, 1H), 1.34-1.16 (m, 8H), 0.85 (t, *J* = 7.5 Hz, 3H), 0.78 (t, *J* = 7.0 Hz, 3H). HRMS (ESI/Q-TOF, m/z): Calcd for $[M]^+$ $C_{61}H_{46}N_4O_3^+$: 882.3564, found: 882.3547.

3. NMR Spectra

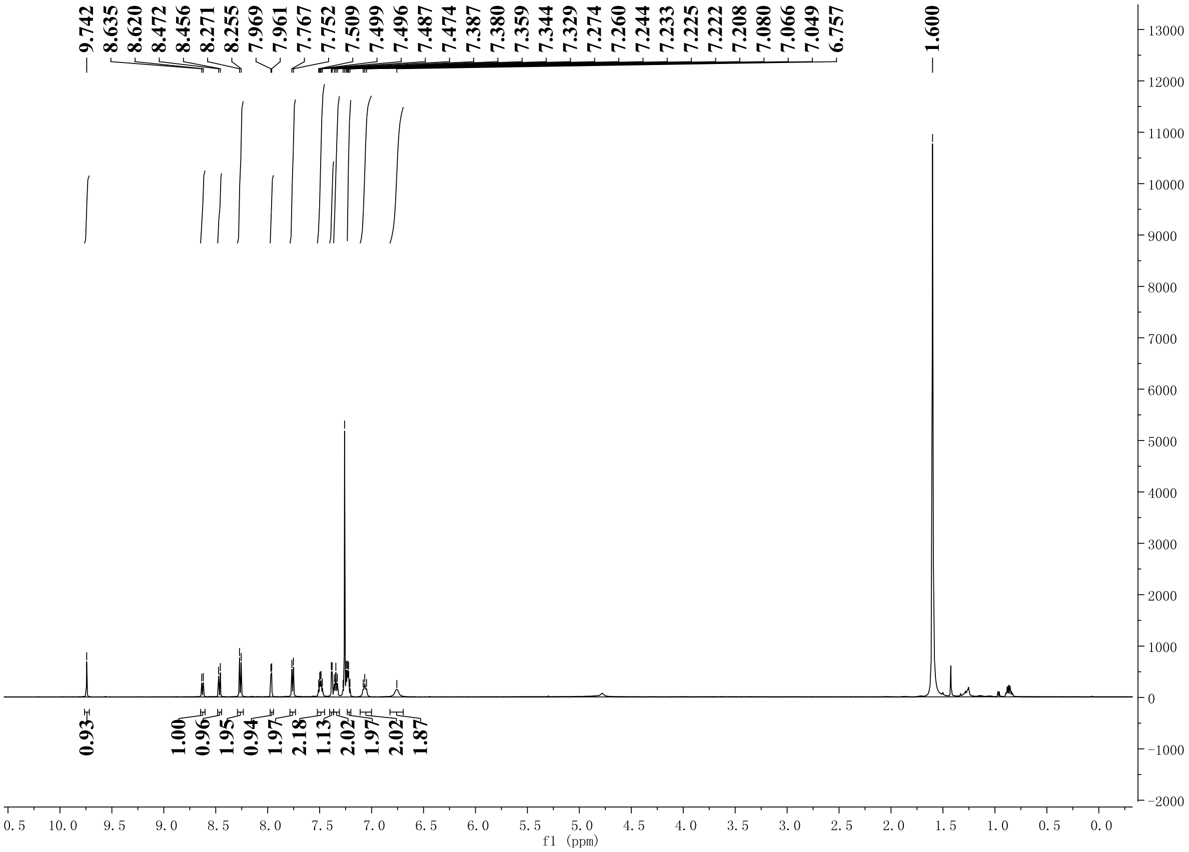

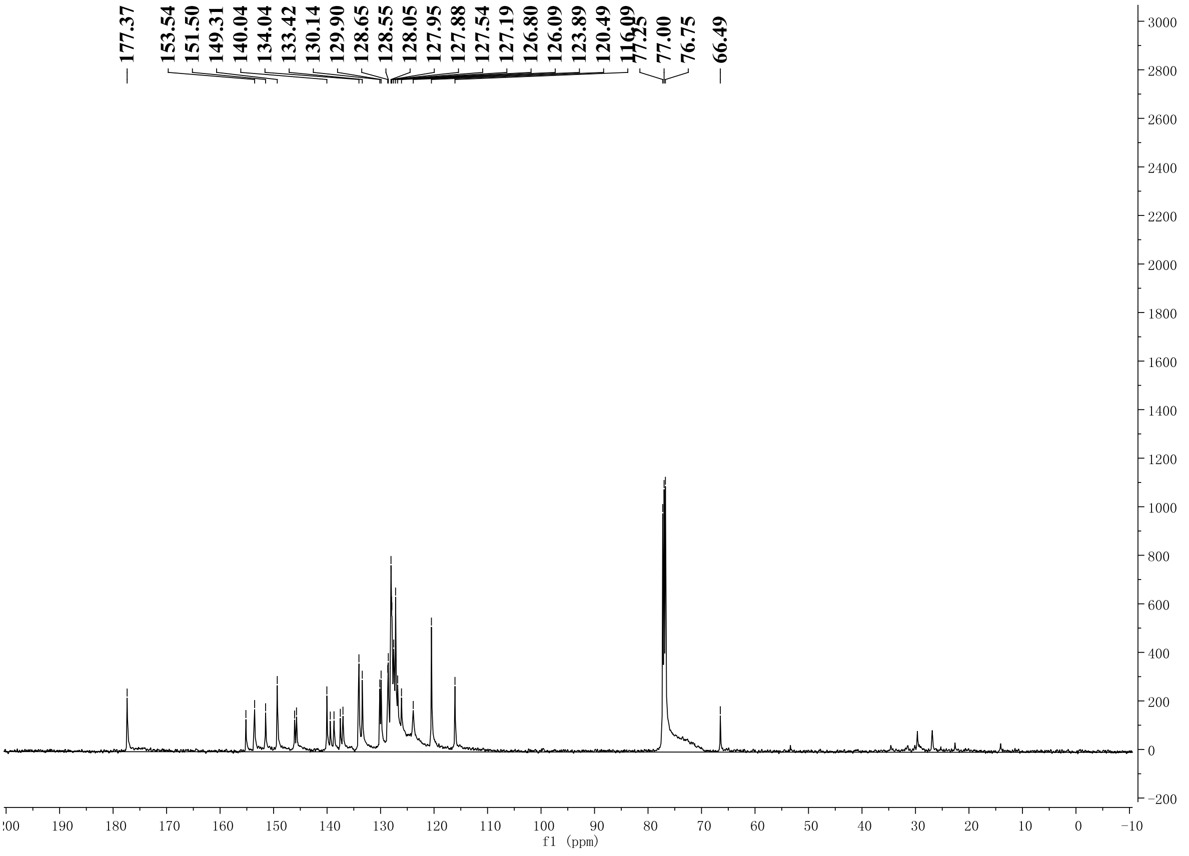

Figure S1. ^{1}H and ^{13}C NMR of **2**.

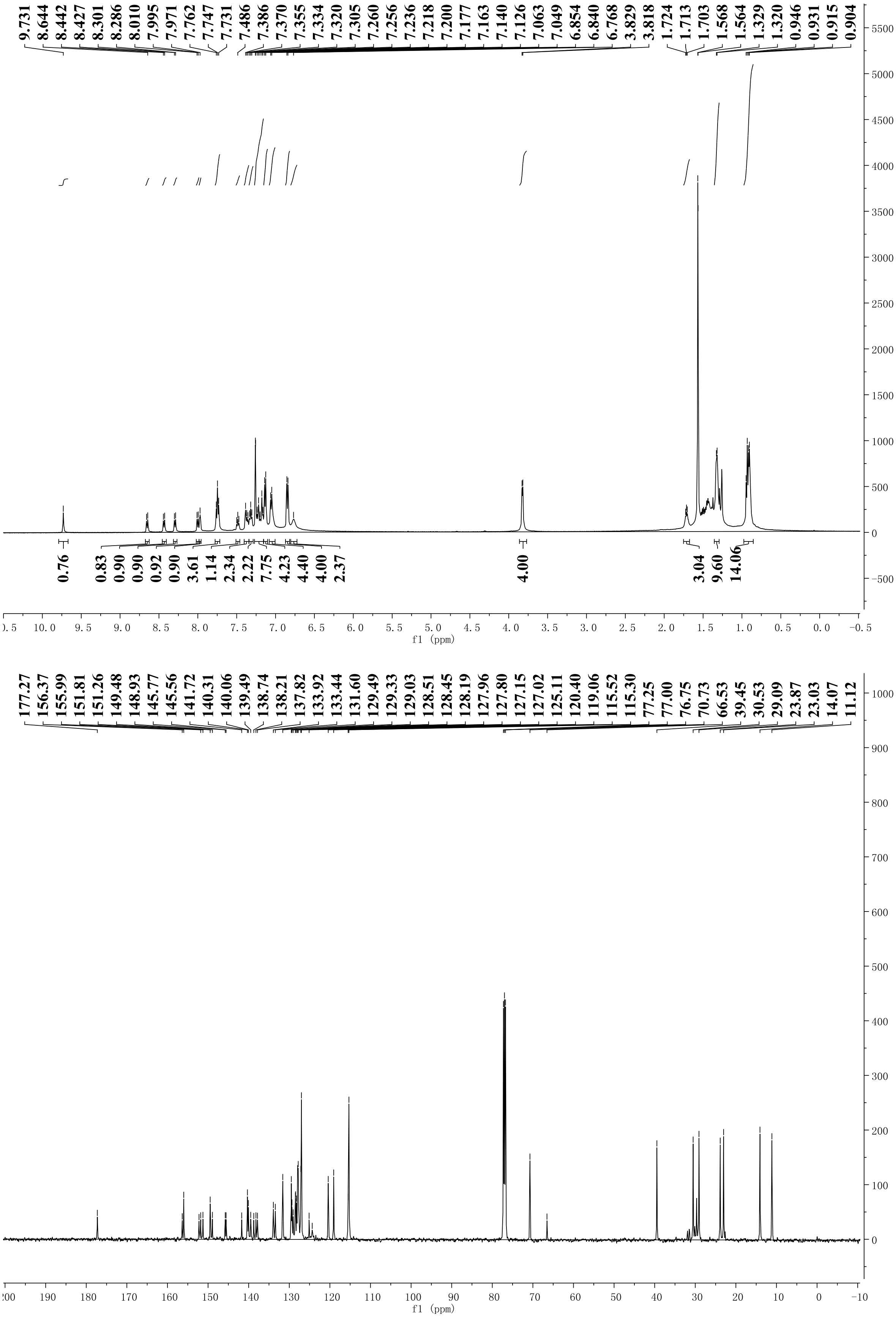

Figure S2. ^{1}H and ^{13}C NMR of **3a**.

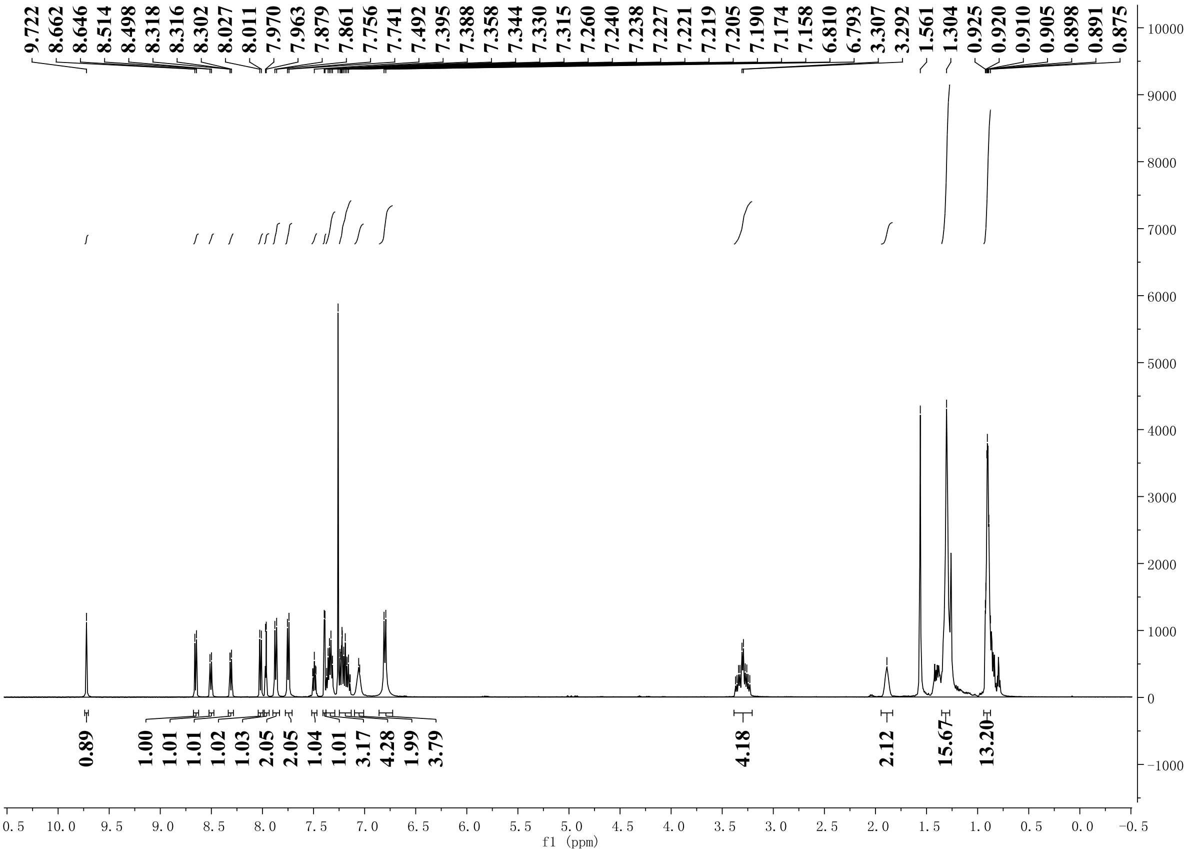

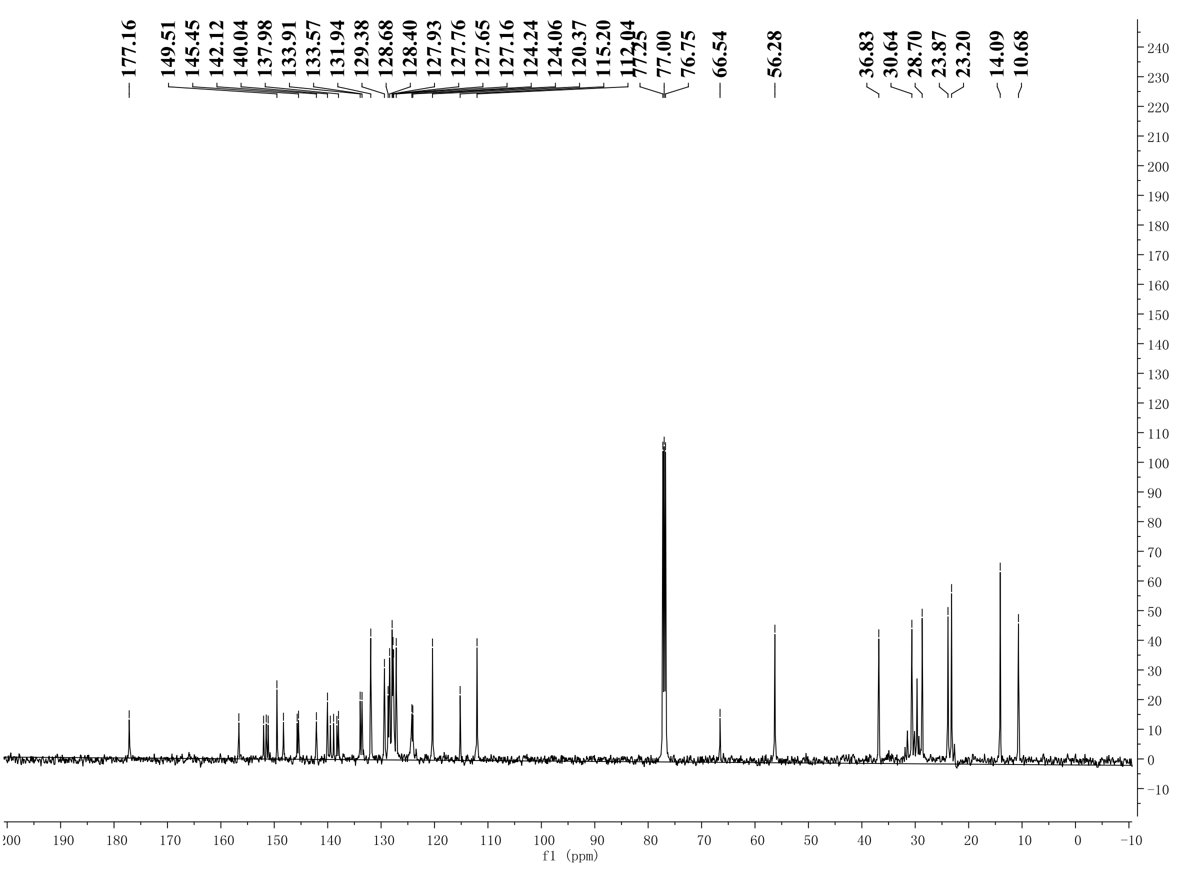

Figure S3. ^{1}H and ^{13}C NMR of **3b**.

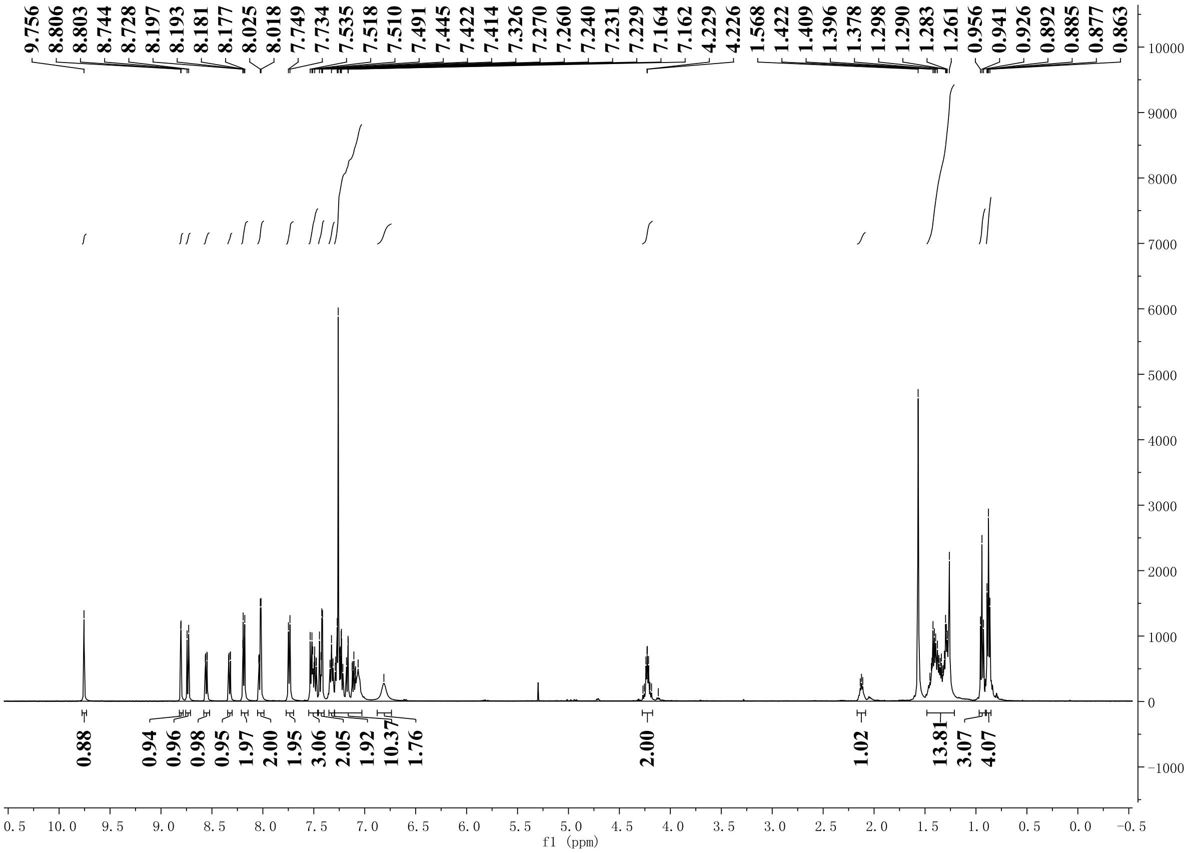

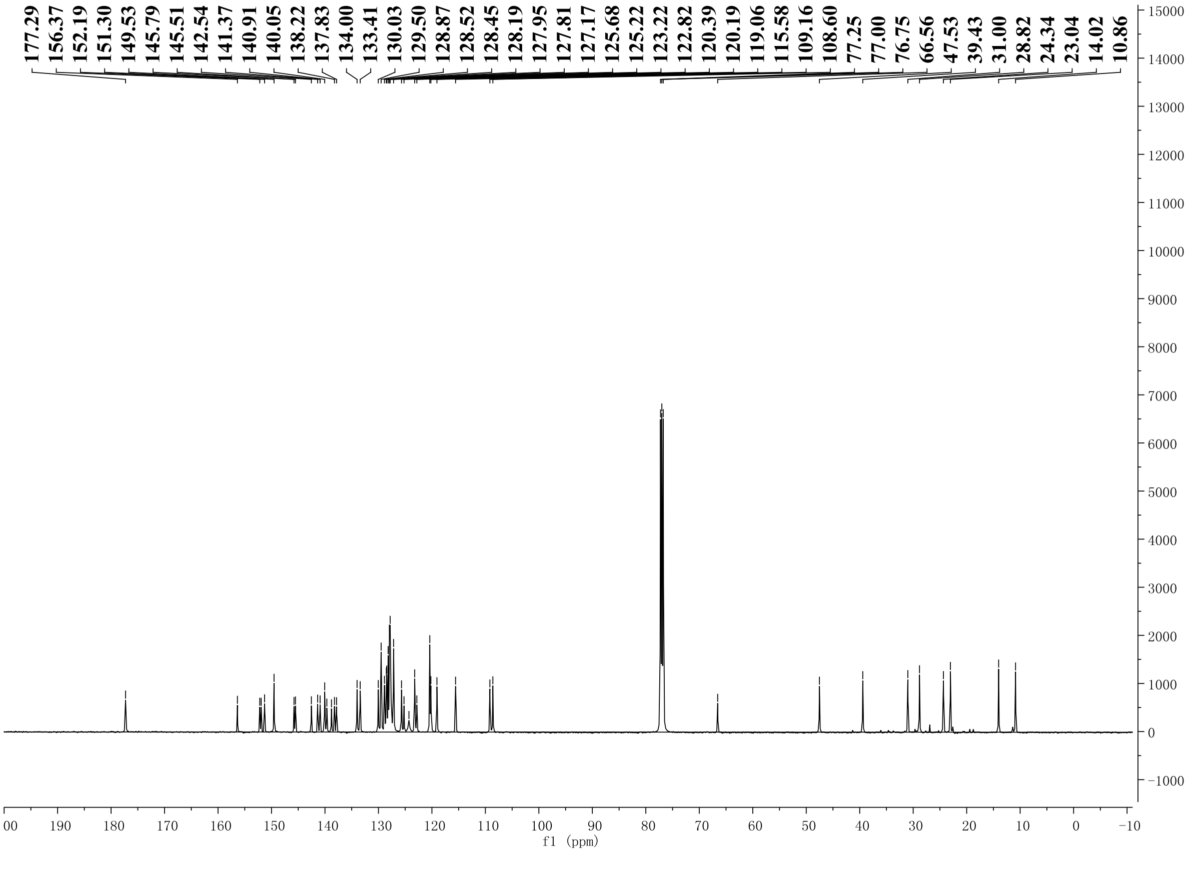

Figure S4. ^{1}H and ^{13}C NMR of **3c**.

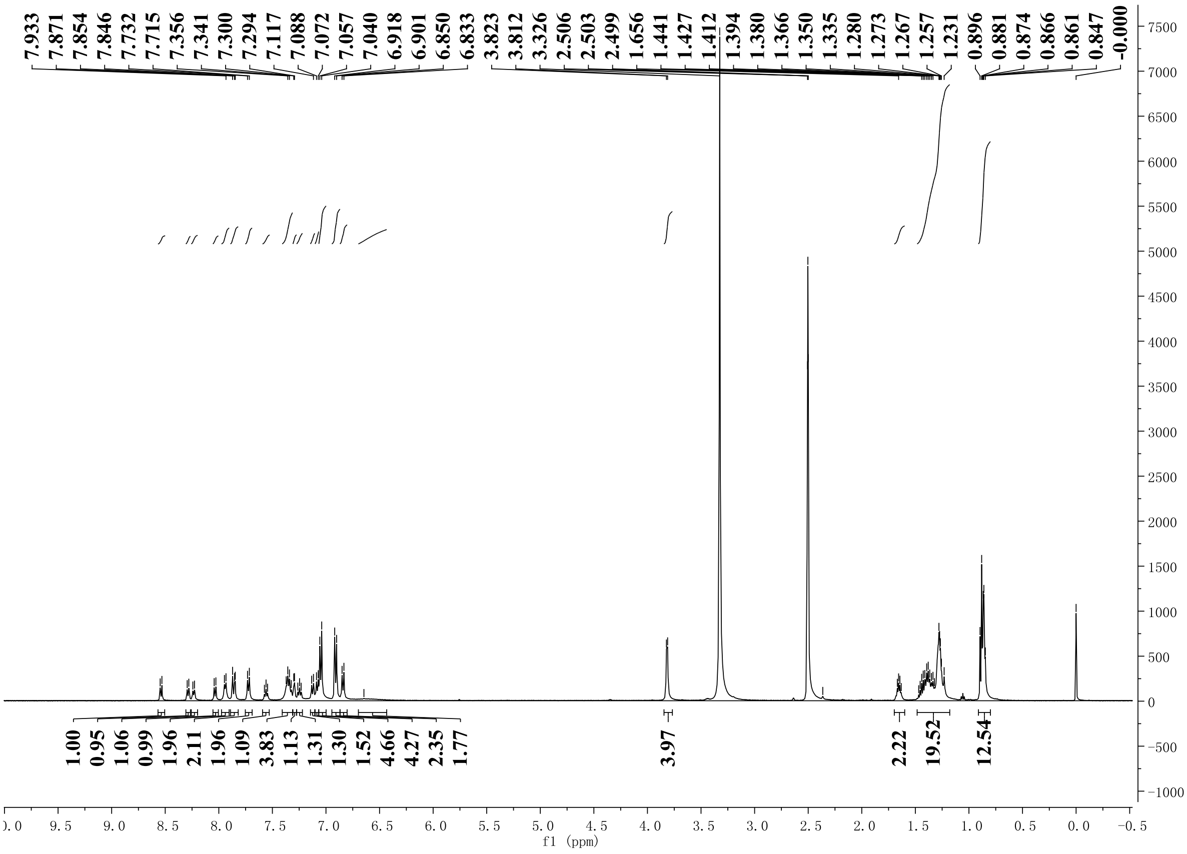

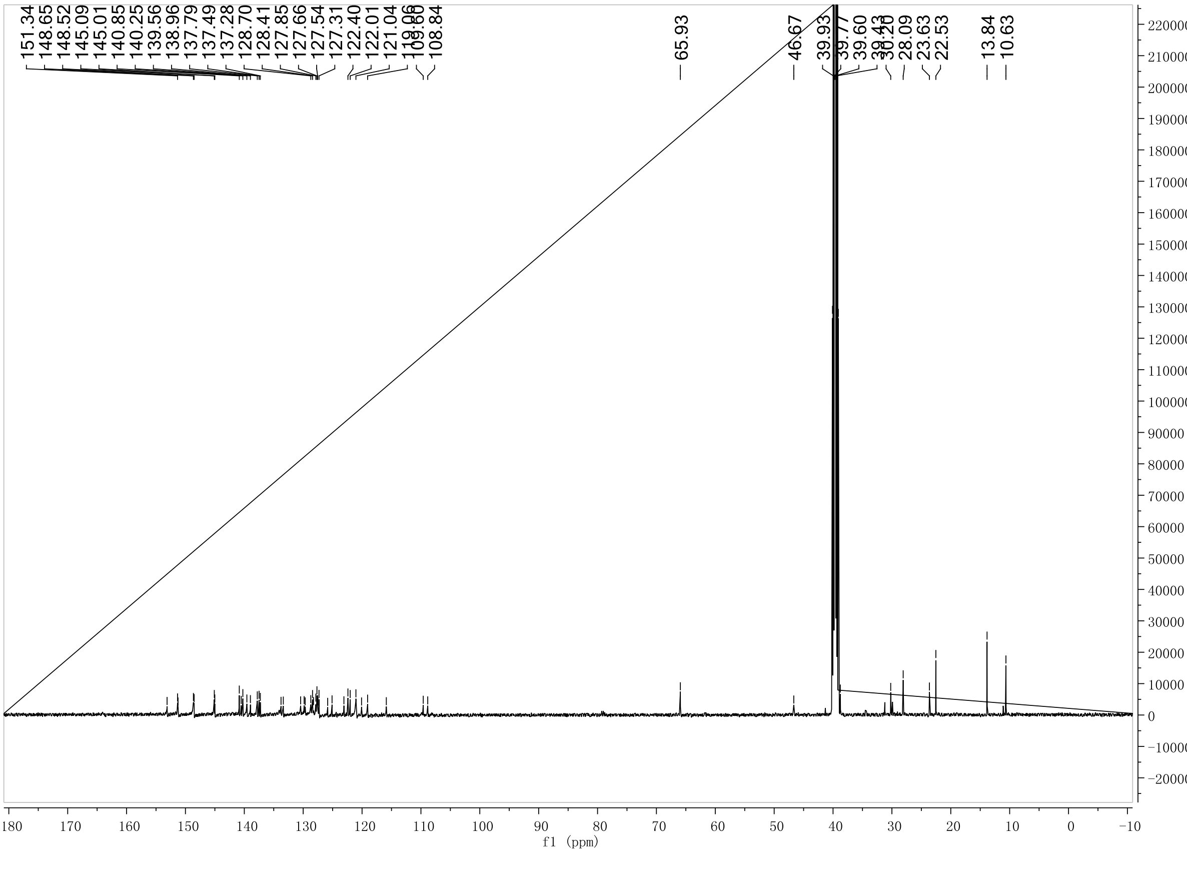

Figure S5. ^{1}H and ^{13}C NMR of **FHD4-1**.

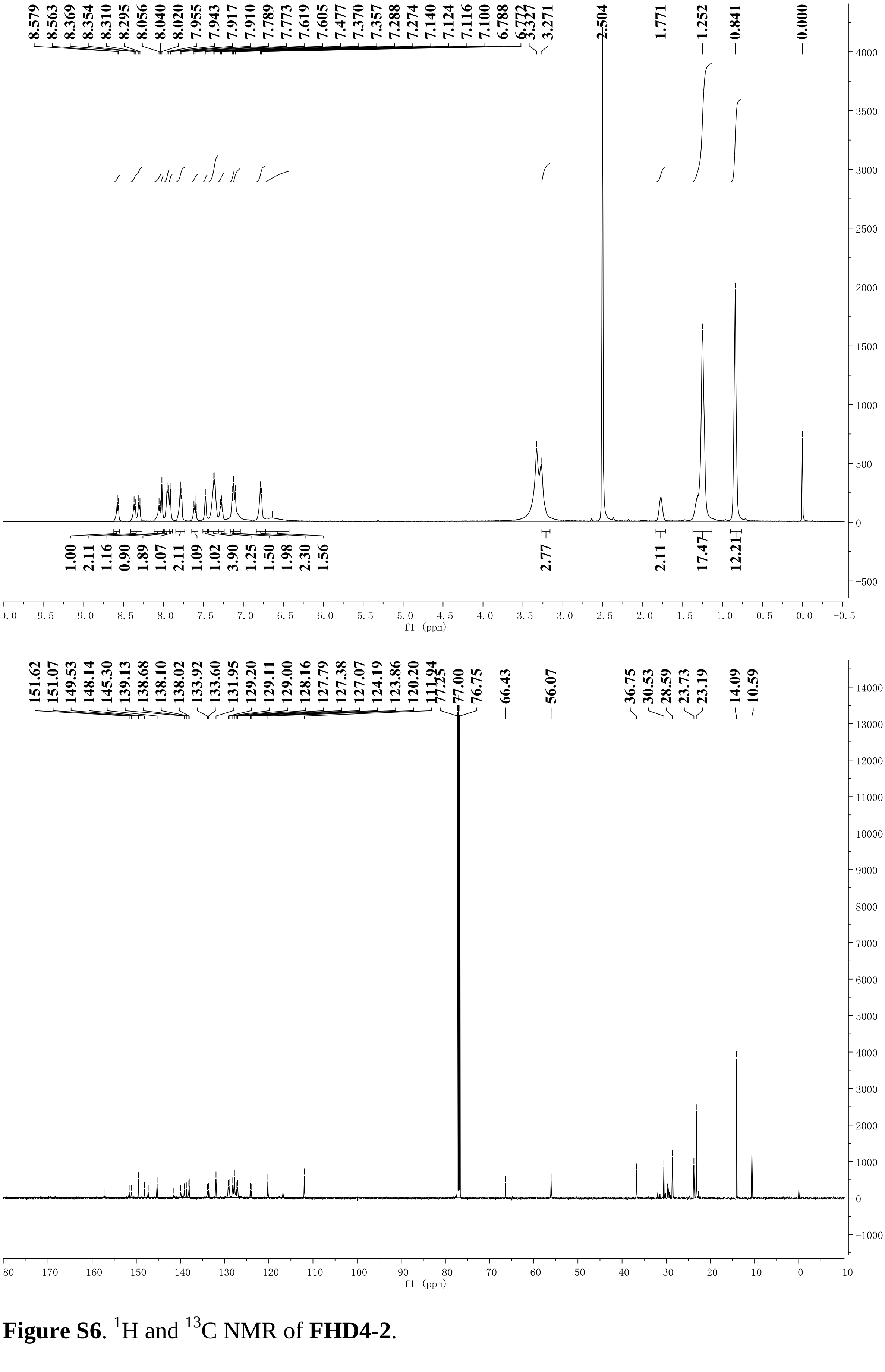

Figure S6. ^{1}H and ^{13}C NMR of **FHD4-2**.

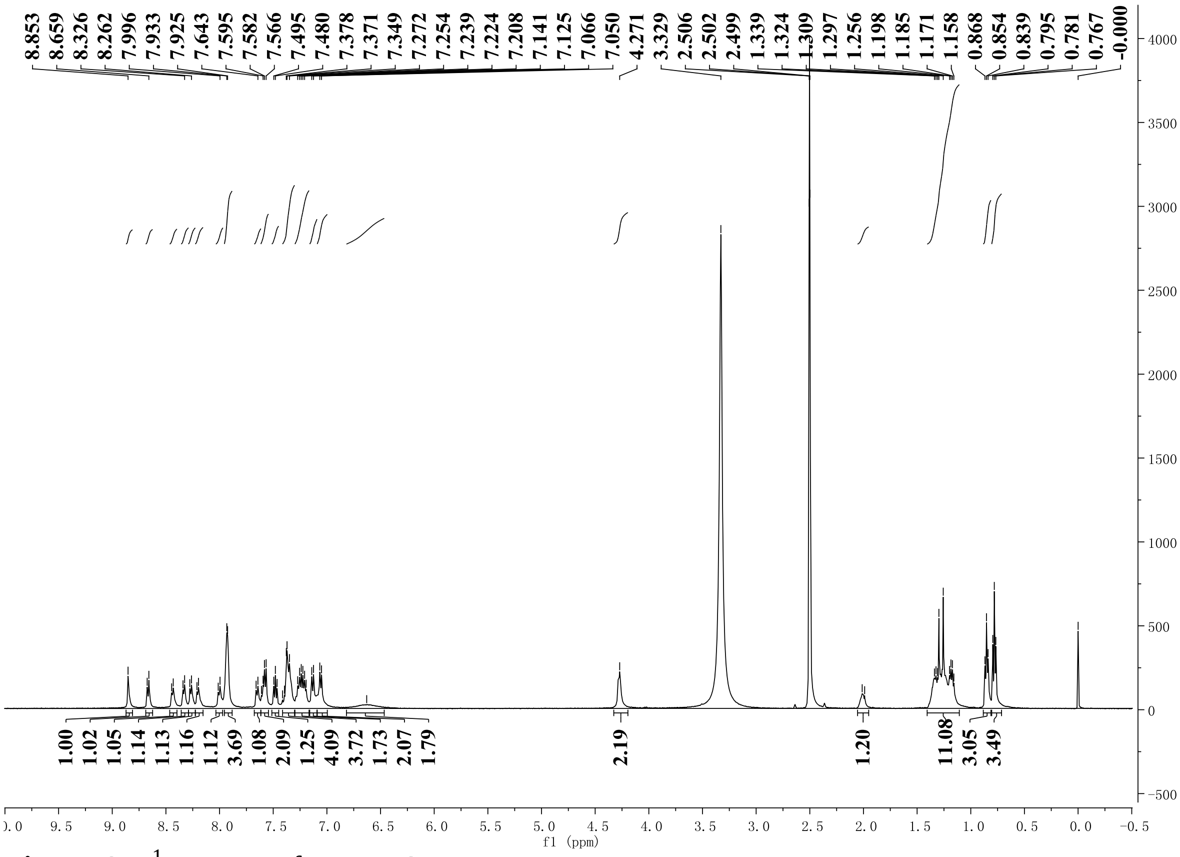

Figure S7. ^{1}H NMR of **FHD4-3**.

4. Mass Spectra

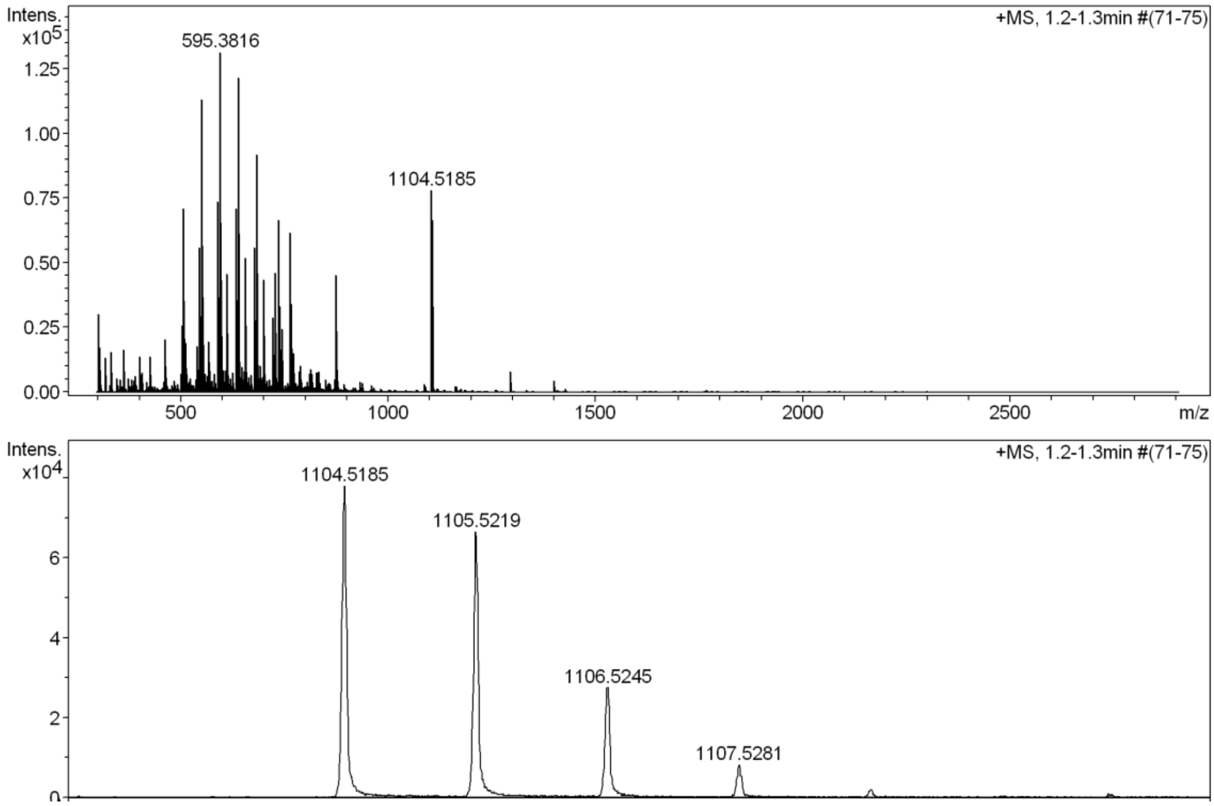

Figure S8. HRMS of **FHD4-1**.

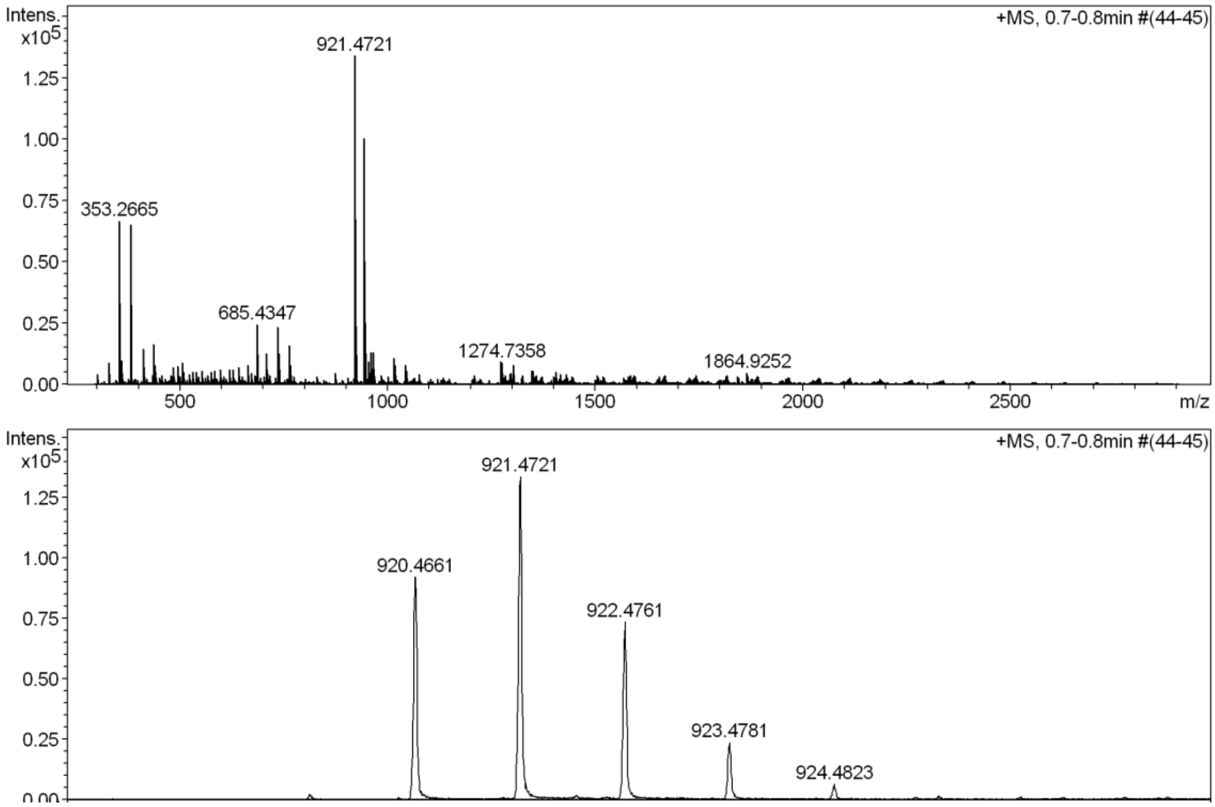

Figure S9. HRMS of **FHD4-2**.

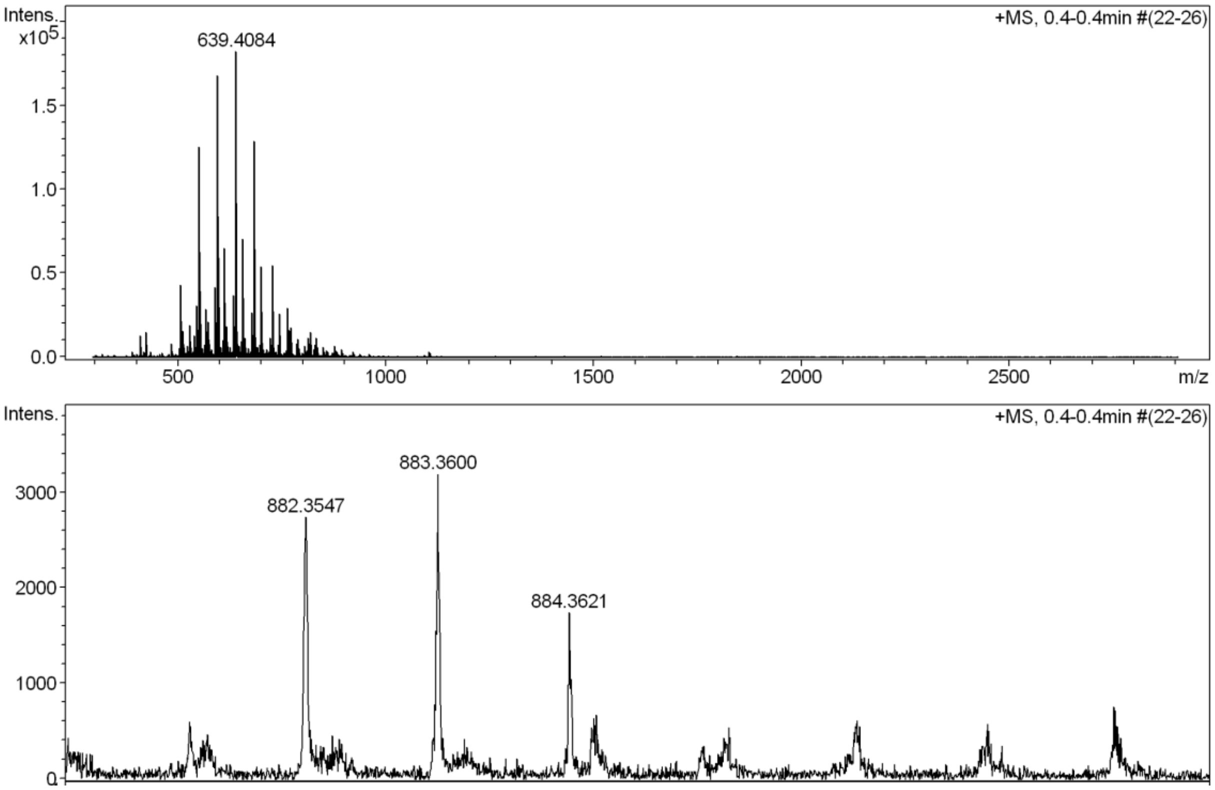

Figure S10. HRMS of **FHD4-3**.

5. Fabrication of DSSCs

Commercial TiO_2 electrode OPV-TiO2-E (Yingkou OPV Tech New Energy Co., Ltd) was adopted and heated at 80 °C for 20 min before using. TiO_2 electrodes were immersed in dye solutions in CH_2Cl_2/MeOH (9:1) (0.3 mM dye) for 24 h. Then the electrodes were flushed with EtOH and dried in air for use. Liquid electrolytes, which consists of LiI (0.1 M), I_2 (0.05 M), 4-tert-butylpyridine (0.5 M) and 1,2-dimethyl-3-propylimidazolium iodide (0.6 M) in acetonitrile/ 3-methoxypropionitrile (1:1), was added dropwise onto the electrodes. The counter electrodes and working electrodes were sandwiched to be dye-sensitized solar cells without mask for the measurements.

6. Supporting References

[S1] M. Xu, X. Hu, Y. Zhang, X. Bao, A. Pang, J. K. Fang, *ACS Appl Energy Mater* **2018**, *1*, 2200-2207.
